# Life after Cell Death—Survival and Survivorship Following Chemotherapy

**DOI:** 10.3390/cancers13122942

**Published:** 2021-06-11

**Authors:** Tamara Mc Erlain, Aileen Burke, Cristina M. Branco

**Affiliations:** Patrick G Johnston Centre for Cancer Research, Queen’s University Belfast, Belfast BT9 7AE, UK; tmcerlain01@qub.ac.uk (T.M.E.); aburke22@qub.ac.uk (A.B.)

**Keywords:** chemotherapy, metastasis, co-morbidities

## Abstract

**Simple Summary:**

Treatment of aggressive cancers often relies on chemotherapy. This treatment has improved survival rates, but while effective at killing cancer cells, inevitably it also kills or alters the function of others. While many of the known effects are transient and resolve after treatment, as survival rates increase, so does our understanding of the long-term health costs that accompany cancer survivors. Here we provide an overview of common long-term morbidities known to be caused by conventional chemotherapy, including the risk of relapse, but more importantly, the cost of quality of life experienced, especially by those who have cancer in early life. We aim to highlight the importance of the development of targeted therapies to replace the use of conventional chemotherapy, but also that of treating the patients along with the disease to enable not only longer but also healthier life after cancer.

**Abstract:**

To prevent cancer cells replacing and outnumbering their functional somatic counterparts, the most effective solution is their removal. Classical treatments rely on surgical excision, chemical or physical damage to the cancer cells by conventional interventions such as chemo- and radiotherapy, to eliminate or reduce tumour burden. Cancer treatment has in the last two decades seen the advent of increasingly sophisticated therapeutic regimens aimed at selectively targeting cancer cells whilst sparing the remaining cells from severe loss of viability or function. These include small molecule inhibitors, monoclonal antibodies and a myriad of compounds that affect metabolism, angiogenesis or immunotherapy. Our increased knowledge of specific cancer types, stratified diagnoses, genetic and molecular profiling, and more refined treatment practices have improved overall survival in a significant number of patients. Increased survival, however, has also increased the incidence of associated challenges of chemotherapy-induced morbidity, with some pathologies developing several years after termination of treatment. Long-term care of cancer survivors must therefore become a focus in itself, such that along with prolonging life expectancy, treatments allow for improved quality of life.

## 1. Fight to the Death

In spite of incontestable progress in the development and implementation of refined treatments, traditional chemotherapy, particularly with cytotoxic agents continues to be the standard of care for many patients, including those diagnosed with leukaemia [1], lymphoma [2,3], breast [4,5,6], lung [7], and colorectal [8] cancers.

Aggressive cancers are those that proliferate fast, disseminate easily and resist treatment, and when cancer cells escape the subtle traps of highly specialised, targeted and refined interventionsƒ drastic measures, such as conventional chemotherapy, come into play. More aggressive cancers or subtypes of cancer primarily affect young patients, and children, adolescents and young adults typically receive the most aggressive treatment regimens, a result of typically faster progressing disease combined with greater regenerative potential, which means this patient demographic will presumably recover faster from adverse effects. The intense contest against cancer cells therefore sets the stage for life-long battles. Where chemotherapy is inevitable, it frequently results in prolonged multimorbidity. It is, after all, a fight to the cell death.

Classic cytotoxins such as anthracyclines, platinum drugs or taxanes are extremely effective in eliminating cells with high proliferation rates, and as such, cancer cells are primary targets [1,9]. Interference with topoisomerase II (anthracyclines) or with microtubule recycling (alkaloids) leads to the arrest of cell cycle progression and apoptosis. Side effects downstream of such therapies are severe and widespread [4,10] for two reasons: (1) these compounds are systemically distributed, and (2) essential somatic cells also include rapidly dividing cells, e.g., the gastrointestinal lining and immune cells.

Inescapably, a proportion of healthy cells are killed during chemotherapy treatments, compromising convalescence. While acute adverse reactions mostly reverse with termination of therapy, others have late onset and persist long beyond drug removal. 

This review will focus on the off-target effects of conventional chemotherapy, and the implications of somatic cell adaptations to cancer fighting drugs.

## 2. Relapse

Metastatic disease is the main cause of mortality for cancer patients [11,12,13]. Improved survival rates have exposed an increased associated risk of distant recurrence in patients treated with chemotherapy, as described in breast, lung, and liver sites [14,15,16,17,18,19]. 

While it is well-known that cancer cells frequently have an intrinsic predisposition to resistance, it is also documented that they are much more likely to acquire resistance following repeated or prolonged exposure to chemotherapy [20]. Cancer cells acquire resistance due to a number of factors including changes in DNA repair mechanisms, limitations in the uptake of chemotherapeutics agents, as well as the active efflux of agents out of the cell, such as via ATP-binding cassette (ABC) transporters [21]. 

Resistance to chemotherapy is usually concomitant or closely followed by the development of gene expression signatures associated with the epithelial to mesenchymal transition (EMT), such as SNAIL, SLUG, and TWIST [22]. Anthracycline treatment is associated with increased invasiveness and multidrug resistance. This has been recently shown to correlate with increased P-glycoprotein-mediated tyrosine-phosphorylation of Annexin A2 [23], which is typically overexpressed and activated in drug-resistant, invasive cell lines [24,25]. Additionally, such resistant cell lines have increased adhesive ability compared with their drug-sensitive counter parts, and more easily attach to the basement membrane of foreign organs following dissemination [26], which significantly improves their colonization success. 

Metabolic rewiring is an often-overlooked consequence of cytotoxic stress, underlying resistance and consequently, relapse [27,28]. In triple negative breast cancer (TNBC), neoadjuvant-driven metabolic shifts to oxidative phosphorylation correlated with enrichment of chemo-resistant cancer stem cells [29], increasing the risk for late relapse. Similar metabolic preference has been found in other cancer types, such as human acute myeloid leukaemia [30]. The relationship between metabolic reprogramming and mitochondrial function has recently been underscored by a seminal study elucidating the mechanisms underlying resistance to mitomycin C in bladder cancer, illustrating that both the tumour and context-specificity impact on the success of cytotoxic intervention [31].

Tumour-free organs are primed by a myriad of tumour-derived factors before or during metastatic colonization. Soluble (cytokines, mitogens) or extracellular vesicle (EV)-transported factors (RNA, proteins) generate a pre- or pro-metastatic microenvironment, typically characterised by increased inflammation, immunosuppression, metabolic and genetic reprogramming, as well as increased angiogenesis and vascular permeability [32]. Increased pro-metastatic EV are seen in chemotherapy-treated melanoma, breast or gastric malignancies [19,33,34,35], and their effects on secondary organs include remodelling of extra-cellular matrix, activation status of resident cells (e.g., inflammatory secretome, T-cell metabolism, angiogenesis), which all contribute to smooth resettling and colonization [36,37,38]. 

While the primary tumour cells have been assumed to be the main drivers of this phenomenon, recent studies implicate stromal cells as significant contributors to the pro-metastatic effects of chemotherapy [39]. The responses and behaviour of neighbouring or infiltrating non-tumour cells determine disease outcome: chemoresistance in glioblastoma, for example, has been shown to rely on the adaptive responses of the endothelial–mesenchymal transition [40]. 

Triads of associated macrophages, tumour and endothelial cells (tumour microenvironment of metastases, or TMEM) are now well-known indicators of poor prognosis in breast cancer, as these are associated with increased intravasation efficiency; a higher density of TMEM directly correlates with increased metastases, both in animal models and patient samples [41]. Density of TMEM foci significantly increases after paclitaxel treatment [15]. Chemoresistant breast cancer cells also appear to induce a specific inflammatory phenomenon that offers increased evasion to senescence and metastatic success [42]. This occurs due to enhanced CXCR2 signalling, either by overexpressing the receptor and thus evading taxol-induced apoptosis [43], or downregulating the receptor while overexpressing ligands CXCL6 and CXCL8 [42]. 

Disseminated tumour cells (DTC) are typically insensitive to chemotherapy, irrespective of cell cycle [44], and are further protected by the perivascular niche they occupy [44,45]. Chemotherapy can further promote DTC dormancy [46], effectively shifting the risk/benefit ratio associated with this therapeutic strategy by increasing the risk for the main cause of cancer-associated mortality, that is, distant metastatic disease [12,47].

Given that most chemotherapy is administered intravenously, endothelial cells (EC) are the first to come into contact with drugs, and the effects on the vascular system are manifold [36,37,38]; predictably, vascular damage is a major consequence of chemotherapy. Exposure to chemotherapy leads to endothelial activation [48,49] and progenitor-mediated regeneration [14], as well as decreased barrier function due to cell death and/or changes in adherens and intercellular junctions [16,36,48]. EC form a continuous but very heterogeneous organ, with unique functional and metabolic profiles, which responds to stress and inflammation in an organ-specific manner [50]; this may actually underlie therapy-driven metastatic organotropism. Most studies investigating vascular damage in response to prolonged exposure to chemotherapy use HUVECs as their model, which does not address the impact on organ function in light of microvascular heterogeneity [51]. For example, expression of genes involved in substrate transport, ECM remodelling, barrier function and endothelial activation, are significantly altered following doxorubicin exposure [52], but their baseline expression varies significantly across the microvascular EC of the brain, lung and heart [50]—the first two are common metastatic organs for TNBC [53], and the cardiac microvascular EC perform essential roles in cardiac muscle function [54]. Indeed, changes in expression of these genes is affected by inflammation in an organ-specific manner, underscoring the markedly different expression profiles [55] and adaptations within each organ, suggesting also an organ-specific response of tumour-free tissues to chemotherapy-induced stress, which could underpin metastases caused by treatment.

The brain, a relatively unlikely site for secondary cancer [13], develops metastatic tumours at a higher rate in patients that receive dose-dense chemotherapy, such as those with lung [56], melanoma [57], colon [58] or TNBC [4,13,53,56,59,60,61,62]. Brain metastasis dramatically curtail life-expectancy and have a severe impact on quality of life. Recent work has revealed specific properties of brain-adapted tumour cells [56], as well as potential microenvironment parameters (e.g., metabolic shifts, resident cell activation) that can support the development of secondary tumours in this tissue [53]. For example, Taxol-primed astrocytes provide contact-dependent support to lung and breast cancer cells via the upregulation of survival proteins GSTA5, CCL2L1 and TWIST, underscoring that somatic cell responses to therapy result in protection of the target (tumour) cells [63]. Similarly, endothelial expression of VEGFR-1 following paclitaxel and cisplatin treatment have been shown to potentiate pulmonary metastasis in a colon cancer mouse model, as a result of improved adhesion of tumour cell to the secondary site [16].

**Figure 1:**
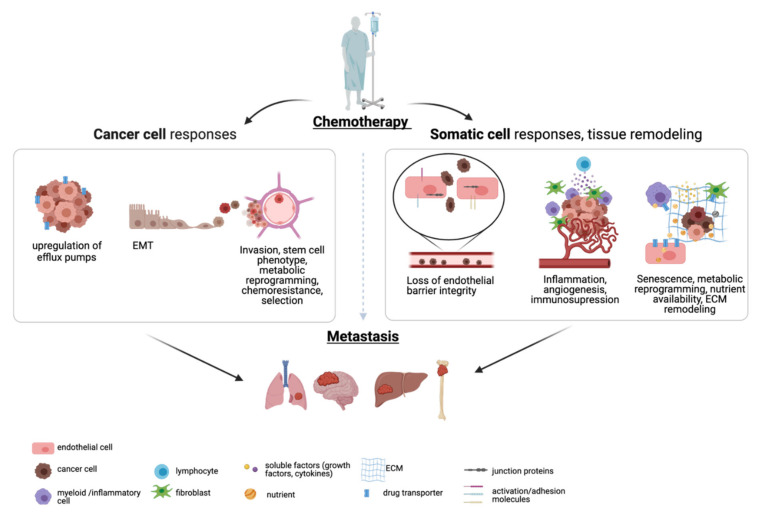
Pathways to Relapse. Chemotherapy-induced relapse includes intrinsic cancer cell resistance, reprogramming and selection, and pro-metastatic changes downstream of somatic cell responses. Cancer cell resistance include overexpression of drug efflux pumps, to actively transport cytotoxic chemicals out of the cancer cell, increased EMT, invasion and adhesion properties, and secretion of extracellular vesicles that prime distant organs (left). Somatic cells in the tumour microenvironment or at distant, tumour-free organs undergo changes in response to chemotherapy. Vascular dysfunction has been shown to contribute to the risk of metastasis and relapse, due to the loss of barrier integrity and resulting vessel leakiness, increasing endothelial activation and a more inflammatory, pro-tumour microenvironment. Chemotherapy also rewires secretory profiles and metabolic preferences, changes in nutrient and substrate availability, which can play into cancer-specific adaptations, often potentiating proliferation and colonisation at secondary sites. Created with BioRender.com (accessed on 5 June 2021) [69].

While microvascular cell plasticity ensures that metabolic demands are met at specific organs and in specific contexts [64], their adaptive responses to stress can involve structural, angiogenic, intercellular communication and metabolic changes likely to favour dissemination and proliferation of circulating tumour cells (CTC) [48,51,65,66,67]. In turn, CTC have been shown to more efficiently evade cytotoxic treatments, as a result of enhanced DNA repair mechanisms [68]. Thus, the combination of innately chemoresistant CTC with compliant microenvironments is bound to heighten the likelihood of developing secondary tumours (Figure 1).

The mechanisms driving resistance in tumour cells, however, are the same as those that permit the survival of non-tumour cells, including the increased expression of drug transporters. ABC transporters are also upregulated in non-cancer cells following exposure to doxorubicin [52]. Cell- and tissue-specific responses to treatment should therefore be better understood to pre-empt and prevent relapse.

## 3. Unintended Casualties

Beyond the risk of metastasis, co-morbidities associated with off-target effects of chemotherapy are increasingly being reported [9,70,71]. Whilst primarily targeting cancer cells because of their high proliferation rates, chemotherapy will result in the death of non-malignant cells, many of which also need replacing at regular intervals, and this results in acute and widely reported effects such as gastrointestinal pathology, hair loss, immune deficiency or neutropenia [72].

Cardiomyopathy is one of the most common persistent complications of chemotherapy [73,74,75,76]. It is influenced by various factors including the drug combination, dosage, route of administration, duration of treatment, as well as patient-specific predisposing factors [76]. A frequently described cause is the accumulation of reactive oxygen species (ROS), which can contribute to both cancer progression or cancer cell death [77], but also underlies several comorbidities associated with treatment, not least heart failure [77,78]. Besides conventional chemotherapy, many classes of drugs have been shown in association with cardiac damage [79] and cumulative dose limits have been set based on their impact on cardiac function. Cardiac damage resulting from chemotherapy varies, with reports of both early onset acute myopericarditis to late onset chronic progressive heart failure [73,80].

The use of cyclophosphamide, for example, is limited due to left ventricular dysfunction between one and ten days following infusion of the first dose [73,80], seen in approximately 28% of patients. Anthracyclines, a family of widely used anticancer antibiotics [81], are the most relevant compounds in promoting heart disease [79]. Because of its powerful anti-cancer effects, doxorubicin has been intensely used in the treatment of aggressive malignancy; however, dosing is conditioned by associated cardiotoxicity, and limited due to cumulative dosing-related arrhythmias, ventricular dysfunction and heart failure [74,82,83]. A retrospective analysis of three clinical trials evaluating cardiotoxicity in breast or small cell lung carcinoma patients treated with doxorubicin, reported that the incidence of chronic heart failure was 4.7% at a cumulative dose of 400 mg/m^2^, 26% at 550 mg/m^2^ and 48% at 700 mg/m^2^ [82]. Evidence of left ventricular ejection fraction decrease [84] can also be observed in 65% of patients who received doxorubicin to treat a childhood malignancy, and 5-year survivors exhibited a 2.4-fold increased risk of developing congestive heart failure [85]. As a result, the recommended cumulative doxorubicin dose has been limited to 450–500 mg/m^2^, but because this is often given in combination with other agents or radiotherapy, there is really no dose that can be considered clinically completely safe [73]. Several mechanisms of anthracycline-induced cardiomyopathy have been proposed, the most common being free-radical mediated oxidative damage to cardiomyocytes, mitochondrial dysfunction and lipid peroxidation [73,75], and ROS mediated autophagy [74].

Epirubicin, an epimer of doxorubicin, has comparable anti-tumour activity but carries much lower (30% less) cardiotoxicity [81], which allows it to be used at significantly higher concentrations [86]. Although the incidence of acute cardiotoxicity drops, the risk of developing chronic heart failure actually increases from 11% after one year to 20% after five years following termination of treatment, and this appears to be due to microvascular EC damage [54] instead of cardiomyocyte death. In arteries treated with doxorubicin ex vivo, EC were observed to peel away from the tunica intima, undergoing apoptosis 7 days after treatment with a sublethal dose [87].

A significant number of EC undergo apoptosis following chemotherapy exposure, particularly the ones found at sites of injection [36,48,88,89,90]. Microvascular EC treated with cisplatin display increased caspase activity with induction of apoptosis and necrosis in the 24 h following treatment [91]. Loss of vascular integrity and barrier function inevitably facilitates extravasation of surviving CTC. EC death in response to chemotherapy has also been proposed to underlie osteonecrosis and osteoporosis; bone vascularization is essential for ongoing dynamic remodelling, regeneration, as well as haematopoiesis [92], and as such, loss of EC will affect perfusion, and compromise the viability of other cells, factors now known to underlie therapy-induced bone loss and increased risk of fracture [92,93].

Microvascular cell death has also been linked to chronic renal failure [36,94], and an increase in EC caspase-3/7 activity [91] has been seen following cisplatin treatment, as well as increased TUNEL-positive renal EC staining as a result of doxorubicin treatment, which occur in a dose-dependent manner [65,67,89]. Chemotherapy-derived hepatic deficiency has also been shown to be associated with somatic, parenchymal and microvascular cell responses [95,96,97].

However, relatively few somatic cells undergo apoptosis upon exposure to chemotherapy, and most of those are replaced upon termination of treatment. 

The cells that survive, adapt.

## 4. What Doesn’t Kill You…

Most tumour cells will die from exposure to therapeutic concentrations of cytotoxins. Most somatic cells will not, and this is the key aspect of the success and reliability of classical chemotherapy [1,9]. Somatic cells are exposed to varying degrees of circulating drugs, and their survival requires that they respond to that stimulus; one of the most common consequences of exposure to chemotherapy (and radiotherapy) is senescence [48,93,98,99]. 

Premature ageing and age-related pathologies develop and persist following chemotherapy, and result in chronic morbidity for many cancer survivors [71]. Irrespective of cancer type, patients treated with chemotherapy often suffer from general malaise such as chronic fatigue, weakness, or unexplained pain, but also frequently, neurocognitive impairment [99]. Premature ageing is seen at physiological, cellular and subcellular/molecular levels, from hearing loss or vision impairment due to cataracts, to telomere shortening or changes in microRNA signalling and epigenetic alterations, as recently reviewed [100]. While predictable in older patients who are also more likely to have pre-existing conditions [101], it is most striking in younger ones, who develop higher risk of severe and life-threatening conditions than the general population and live with those conditions much longer [70]. In some cases, such as in Hodgkin’s lymphoma survivors, excess mortality risk has been shown to persist for >20 years post-diagnosis [93,102].

While associated with age-related diseases, the stable arrest of proliferation brought upon by the process of senescence can be desirable when induced in cancer cells themselves [103], but is associated with relapse when triggered in somatic cells, such as fibroblasts [104] or endothelial cells [48,52,98,105]. Senescent mesenchymal stem cells promote breast cancer proliferation and invasive behaviour via secretion of IL-6 [106]. Chemotherapy-induced senescence is also associated with bone loss [93,102] by directly affecting osteoblast function and dynamic bone homeostasis [93], in fact, to a much larger extent than oestrogen-based treatments. Indeed, chemotherapy-induced bone loss has been shown to be rescued by selective removal of senescent osteoblasts [93].

Senescence-associated secretory phenotype (SASP) is a pro-inflammatory response, which is necessary during tissue regeneration and wound healing [104], but can also drive chronic wounds, angiogenesis and pro-tumorigenic microenvironments [107]. SASP is a common consequence of exposure to chemotherapy, and is beneficial in pre-malignant lesions [98,104]: senescence-induced angiogenesis has been shown to allow more efficient drug-delivery because of better tumour perfusion [98]. In a KRAS mutant model of pancreatic adenocarcinoma, the induction of senescence further resulted in sensitization to PD-1 checkpoint blockade downstream of SASP-induced immune infiltration [98]. Conversely, the inhibition of senescent-associated signalling has also provided opportunities for refinement of cancer therapy and improved outcomes [93]. In prostate cancer, inhibition of stromal cell-derived, senescence-induced amphiregulin prevented chemoresistance and improved immunocompetency [108]. In acute myeloid leukaemia, cancer cell senescence has been shown to select for stem-cell properties associated with increased risk of relapse [109]. While the effects of senescence are specific to the cell type and context within different cancers, for the most part, the proinflammatory and angiogenic aspects associated with this process promote tumorigenesis and prolonged side effects from treatment. 

When exposed to the diluted concentrations found in capillary beds, microvascular EC encounter sublethal doses of chemotherapy, and while this does not cause EC cell death, a significant proportion of these cells undergo senescence [91,110,111]. The consequences of this are unique to the endothelium, which has a vessel-preserving profile that includes transient instead of persistent IL-6 activation [112]. While the effects on metastatic success are still unknown, this response and its impact on the tissue landscape is bound to be organ-specific [64]. As the impact of EC dysfunction is seen at the root of a myriad of pathologies, from diabetes, atherosclerosis, and ischemia to neurodegenerative conditions [51,64], it is not surprising that many of the co-morbidities associated with chemotherapy are strongly linked to microvascular EC reprogramming, and are reflected not only in the most severe clinical issues associated with chemotherapy, such as cardiac insufficiency, but also some of the most subtle long-term pathologies. 

Reactions at the site of infusion are well known and have been reported in ~45% of patients [48,52], but the mechanisms affecting microvascular networks of distant organs such as the lungs, brain and liver are poorly understood. However, it has been shown that lung microvascular EC cells isolated from mice one week after epirubicin infusion show impaired viability and function, indicating effects that occur and persist in vivo [48]. The exact mechanism of anthracycline-induced endothelial dysfunction is yet unclear, but multiple factors have been implicated in its development; one mechanism is thought to be the inhibition of endothelial nitric oxide synthase (eNOS) as well as increased ROS generation [51,64].

An additional common complication of cancer [113,114] and its associated hypoxic microenvironment is that of dysregulated coagulation pathways [115,116,117]. When compounded by the effects of treatment [37,118], the overall likelihood of cancer patients experiencing thrombotic events is significantly increased. Most drug classes cause endothelial activation, resulting in expression of cell surface proteins involved in intercellular communication and adhesion, to facilitate arrest and traffic of inflammatory and immune cells to sites of injury. Anthracyclines and taxanes appear to work synergistically [66,119,120], exacerbating vascular damage, which prevents the understanding of individual contributions from compounds usually administered as multi-drug cocktails [6,121]. Platinum-based combination treatments significantly increase the risk for vascular ischemic events [122,123], with Raynaud’s syndrome, for example, preceding acute myocardial infarction in young testicular cancer patients [122]. These effects are linked to endothelial activation, as shown by increased expression of von Willebrand Factor associated with occlusive events [124], or increased expression of intercellular adhesion molecule 1 (ICAM-1) and fibrinolysis-associated factors in EC after cisplatin and cyclophosphamide treatments [49]. EC death following cisplatin exposure is also associated with release of procoagulant endothelial microparticles, further contributing to hypercoagulability [125].

Overall, the incidence of thromboembolism is 15% in patients who receive chemotherapy, compared to 2.5% in the general population [126]. Baseline risk of thrombosis in breast cancer patients, for example, increases from 1% to 6.8% following chemotherapy [127], and to a striking 17.6% frequency if patients receive a (not unusual) five-drug treatment regimen [128].

Exacerbated local and systemic inflammation [129] results from the combined effects of EC activation [130,131], but also from widespread tumour and non-tumour cell death [131,132,133]. Reciprocal and multidirectional effects of cell death, vascular activation, tumour-secreted factors, and therapy-induced responses, such as senescence, promote a widespread state of inflammation [134]. The effects of chemotherapy on intestinal epithelium, an inevitable target due to its high turnover rate, results in massive endotoxin release from gut microflora into circulation [135]. Inflammatory mediators, in turn, further contribute to microvascular remodelling, activation and increased permeability, perpetuating the cycle of vascular pathology, cardiovascular disease and oedema [136], as well as affecting the response to treatment [137] and potential for relapse [99].

The development of neurological disease and cognitive impairment is one of the most common aspects of morbidity seen in patients treated with conventional chemotherapy, and results from the combination of all of the aforementioned effects of treatment: chronic inflammation, oxidative stress, neuron death [131,138] and microvascular alterations [131]. A pre-clinical study in mice found that CNS progenitor cells and oligodendrocytes are susceptible to clinical doses of 5-Fluorouracil (5-FU) [138], and that the neurological effects of this anti-metabolite actually worsened over time due to progressive demyelination [139]. Cognitive impairment can develop either during or post-treatment, with varying degrees of severity and reversibility [139]. Memory loss, disorganized thoughts, difficulty concentrating, and declines in recall and processing speed are seen in up to 80% of chemotherapy patients. While generally 35% of these patients report symptoms for years after completion of treatment [135], the proportion of breast cancer patients reporting long-term effects is almost twice that, nearing 60%, particularly in those treated with doxorubicin and/or cyclophosphamide [131,138]. A recent study has shown that these effects persist for 20 years after treatment, and those with lower cognitive function had associated higher levels of inflammatory markers [140]. These side effects correlate with structural changes in the brain, including a significant decrease in areas of grey and white matter [135,141]. In mice treated with paclitaxel, activation and time to resolution of circulating cytokines (CXCL1, Il-1b, and TNF-α) showed a transient pattern, with CXCL1 persisting for longer [134]. Neuroinflammation was shown to be concomitant with lowered microglia reactivity, and interestingly, expression of individual neuroinflammatory marker was specific to individual brain regions [134].

Endothelial dysfunction and inflammation in the cerebrovascular system contribute greatly to cognitive impairment, and underlie reactive gliosis with accompanying breakdown of neurovascular function, hypoperfusion [142], neuronal death and vascular dementia [100,143]. 

In turn, peripheral inflammation is known to impact on the integrity of the blood brain barrier (BBB) [144]: increased circulating levels TNF-α, IL-6 and IL-1β from peripheral tissues causes disruption of EC–EC tight junctions. A permeable BBB then results in increased immune cell infiltration and a disproportionate cytokine content within the CNS, promoting a neuroinflammatory response [134,144]. These parameters reported in chemotherapy-treated cancer survivors are typically observed in the context of neurodegenerative disease, seen in patients with Alzheimer’s, Parkinson’s or Multiple Sclerosis, and result in the activation of astrocytes and microglia, defective neuronal regeneration, and increased rates of neuronal death [135]. 

In the context of cancer patients, these effects are frequently compounded by deficient perfusion due to cardiac insufficiency and reduced ejection volume, consequently impacting on systemic oxygenation. Thus, cognitive impairment is one of the most pervasive and persistent effects of cytotoxic therapy and is observed in most patients, irrespective of cancer type or drug combination [71].

Outside the CNS, chemotherapy is also known to affect the peripheral nervous system, and chemotherapy-induced peripheral neuropathy (CIPN) is not uncommon, particularly among patients treated with alkaloids and platinum-based drugs [145], and partially caused by chemotherapy-induced oxidative stress [146]. CIPN has been reported in 30–70% of patients receiving chemotherapy, and in severe cases it results in treatment discontinuation. It causes neuropathic pain, numbness in extremities and oversensitivity to thermal and mechanical stimuli [147], which are directly associated with damage to sensory and motor nerves [147]. It is also seen as a result of alterations in blood perfusion, with symptom severity typically increasing over time [141]. Taxanes, by affecting the dynamics of cytoskeleton components, disrupt intracellular vesicle transport, and thus, the adequate transmission of nerve impulses that are germane to peripheral nerve function [147]. Concurrent with increased astrocyte activation and upregulation of pro-inflammatory cytokines in the CNS, markers of neuronal injury, CCL2 and CCL3, were also seen to be upregulated in peripheral nerves of mice treated with Paclitaxel [144]. Platinum-based drugs are known to contribute to CIPN by DNA damage to neurons and axonal degeneration, whereas drugs affecting microtubule dynamics, such as paclitaxel, cause axonal degeneration via microtubule stabilization and contribute to both loss of cell viability and disrupted intracellular vesicle transport [144].

## 5. Tomorrow’s Problem

**Figure 2:**
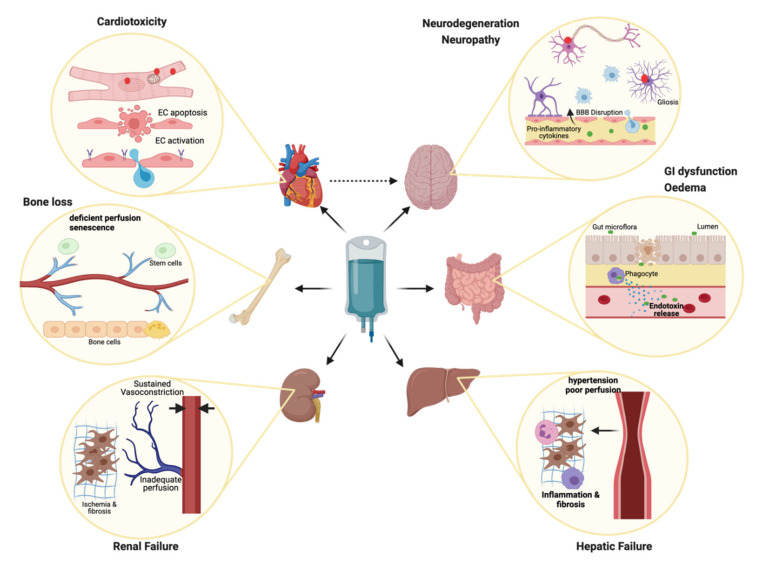
Tomorrow’s problem. Chemotherapy is associated with severe side effects including multi-organ failure. The best characterized side effect of therapy is cardiotoxicity, which can become apparent immediately or in the years following therapy, in severe cases leading to chronic heart failure and death. Endothelial cell apoptosis or activation can lead to increased risk of thrombotic events. Cancer patients also commonly experience chemotherapy-induced cognitive impairment, which may be the result of neurotoxicity, neuroinflammation, BBB breakdown, oxidative stress, and indirectly, cardiac insufficiency. Bone loss due to vascular defects, oestrogen deficiency or resident cell senescence, leads to an increased risk of fractures in patients. Damage to the gut lining and microbiome underlies not only typical nausea and digestive problems, but also systemic inflammation, endotoxin release and subsequent organ damage; Chronic hepatic and renal insufficiency are commonly associated with chemotherapy, and risk for these co-morbidities is compounded by multiple factors, which include loss of vasoregulation and vessel narrowing leading to ischemia, fibrosis and inflammation. After discontinuation of therapy, many of these effects persist or progress, substantially impacting survivors’ quality of life. Created with BioRender.com (accessed on 5 June 2021) [69].

Removal of cancer cells and prolonged life expectancy is often offered in exchange for quality of life. Chronic inflammation, cardiac insufficiency, widespread vascular dysfunction and premature ageing compound life-long health problems. Figure 2 summarises the effects most reported and observed in patients that survive chemotherapy-treated cancer, and how these relate to unintended cell death or essential survival strategies of normal cells that can challenge tissue integrity and organ function. 

The majority of patients diagnosed with highly aggressive cancers are children and young adults, and the trend of cancer incidence in this demographic has shown a staggering 30% increase in the last 40 years [148]; colorectal cancer, for instance, has increased 2% every year since 1994 in patients under 50 years of age [149]. Childhood cancer patients have high survival rates, which in turn, results in either prolonged morbidity and a significant health burden, or late onset of conditions that will compromise their later decades of life [150]. The challenges presented by still insufficient targeted therapies [151], combined with the steady increase in the number of cancer survivors needing care for prolonged periods of time, inflict a significant strain on healthcare systems. 

Following patients over time has allowed the cataloguing and recording of less obvious effects of treatment [152], which in turn, allows research into the mechanisms at the core of those effects. The unique biology of adolescent and young adult cancer [153,154] provides a rationale for the refinement or development of alternative therapies more suitable for use in the treatment of older adults, such that stratification by age at diagnosis [155] has been proposed to address the challenges associated with treatment of these patients. 

The aspects of co-morbidity outlined above provide the strongest argument for personalised medicine, where patient-specific gene signatures and molecular profiling combined with clinical features hold the promise of revolutionising treatment. Deep molecular profiling has allowed predicting the response to, for example, immune checkpoint inhibitor therapy [156], and in one case of advanced prostate cancer, resulted in significantly improved prognosis following successful targeted molecular intervention [157].

However, an abundance of studies and trials have highlighted the difficulties and limitations of translation of personalised medicine based on genetic screens into general clinical practice [158]. In some cases, basing treatment on molecular drivers irrespective of cancer type has offered an advantage over pathological stratification [158], but responses to treatment are inherently context-specific. An illustrative example is that of HER2 targeting in breast cancer, which results in significant improvement in life expectancy [159], whereas targeting the same receptor brings little benefit to treating other HER2+ malignancies, such as gastric cancer [158,160]. Tumour heterogeneity and cancer evolution, often as a result of treatment itself, mean that cytotoxic approaches are likely to remain necessary, at least for now.

Recent studies have also explored the effects of nutrition on improving treatment [161] or the synergistic or additive effects of bioactive compounds in traditional treatment of colorectal cancer [162]. These approaches can help decrease the dose of more toxic treatments, while minimizing collateral effects and prolonged morbidity for cancer patients.

Research into identification of genetic [84,163] and metabolic signatures [164,165,166,167,168], advanced delivery approaches [165,166,167,168,169] and creative, tailored combinations will allow targeting a much smaller and relevant cohort of cells (cancer cells).

## 6. Conclusions

The reliability of conventional chemotherapy compels its use for the foreseeable future; while research will hopefully render this approach obsolete, accessory and sophisticated measures need to be implemented to mitigate, reverse and ultimately avoid collateral morbidity.

Acknowledging the unique nature of the pathology, patient and context are essential to develop the most specific and least damaging treatments, while exploiting intrinsic or induced susceptibilities is what will ultimately improve treatment and life after cancer. 

## Data Availability

Not applicable.
